# Toxic and Repellent Effects of Volatile Phenylpropenes from *Asarum heterotropoides* on *Lasioderma serricorne* and *Liposcelis bostrychophila*

**DOI:** 10.3390/molecules23092131

**Published:** 2018-08-24

**Authors:** Yang Wang, Shanshan Guo, Juqin Cao, Xue Pang, Zhe Zhang, Zhenyang Chen, Yang Zhou, Zhufeng Geng, Yuli Sang, Shushan Du

**Affiliations:** 1 Beijing Key Laboratory of Traditional Chinese Medicine Protection and Utilization, Faculty of Geographical Science, Beijing Normal University, No. 19 Xinjiekouwai Street, Beijing 100875, China; wangyangjs@mail.bnu.edu.cn (Y.W.); guoshanshan@mail.bnu.edu.cn (S.G.); juqincao@mail.bnu.edu.cn (J.C.); pangxue0924@mail.bnu.edu.cn (X.P.); zhezhang@mail.bnu.edu.cn (Z.Z.); sunnychen@mail.bnu.edu.cn (Z.C.); gengzhufeng@bnu.edu.cn (Z.G.); 2 Medical Chemistry Department, School of Basic Medical Sciences, Ningxia Medical University, No. 1160 Shengli Street, Yinchuan 750004, Ningxia, China; 3 College of Pharmacy, Liaoning University, No. 66 Middle Chongshan Road, Shenyang 110036, Liaoning, China; swzyunying@163.com (Y.Z.); ylsang1973@163.com (Y.S.); 4 Analytical and Testing Center, Beijing Normal University, No. 19 Xinjiekouwai Street, Beijing 100875, China

**Keywords:** *Asarum heterotropoides*, toxicity, repellency, phenylpropenes, Lasioderma serricorne, *Liposcelis bostrychophila*

## Abstract

Toxic and repellent effects of the essential oil from *Asarum heterotropoides* Fr. Schmidt var. mandshuricum (Maxim.) Kitag. were evaluated against *Lasioderma serricorne* and *Liposcelis bostrychophila*. The essential oils (EOs) from roots (ER) and leaves (EL) of *A. heterotropoides* were obtained separately by hydrodistillation and characterized by gas chromatography-mass spectrometry (GC-MS) analysis. Major components of ER and EL included methyleugenol, safrole, and 3,5-dimethoxytoluene. Both ER and EL of *A. heterotropoides* showed certain toxicity and repellency against *L. serricorne* and *L. bostrychophila*. 3,5-Dimethoxytoluene, methyleugenol, and safrole were strongly toxic via fumigation to *L. serricorne* (LC_50_ = 4.99, 10.82, and 18.93 mg/L air, respectively). Safrole and 3,5-dimethoxytoluene possessed significant fumigant toxicity against *L. bostrychophila* (LC_50_ = 0.83 and 0.91 mg/L air, respectively). The three compounds all exhibited potent contact toxicity against the two insect species. Here, the EL of *A. heterotropoides* was confirmed to have certain toxicity and repellency against stored product insects, providing a novel idea for the comprehensive use of plant resources.

## 1. Introduction

As Maxmen wrote in “Crops pests: Under attack”, we are faced with a hungry planet and rising temperatures, and pests will be an increasingly part of the story. Insects generally thrive in warmer weather. As the temperature rises, they eat more, mate more, and produce more young, which causes large amounts of crops to be lost to insects [1]. Thus, the threat of insects to agriculture and food is expected to increase. In the warehousing industry, pest control has always been a hot topic since insects damage stored-products, grains, and processed products in a variety of ways. *Lasioderma serricorne* (Coleoptera, Ptinidae) and *Liposcelis bostrychophila* (Psocoptera, Liposcelididae) are common pests of a large number of stored products worldwide, such as grain, food, spices, herbs, and tobacco. They can grow rapidly under warm, moist conditions, thus exerting great pressure on the management of stored items. These insects always cause irretrievable resource wasting and economic losses by consuming and infesting stored products [2,3,4,5,6].

Synthetic contact insecticides, fumigation, and insect repellents, such as pyrethroids—cyfluthrin and deltamethrin—phosphine, methyl bromide, and DEET (*N*,*N*-diethyl-3-methyl-benzamide) have been applied to protect stored crops and products over long periods. However, random use of these conventional insecticides and repellents has triggered severe environmental and health problems [7,8,9,10]. Botanical insecticides have long been thought to be attractive alternatives for pest control because of their safety and eco-friendly properties [11,12,13]. Essential oils (EOs) extracted from natural plants are mixtures of low molecular weight with highly volatile secondary metabolites. Diversity of chemical compositions imbue EOs with diverse pharmaceutical and biological activities. Contact, fumigant insecticidal effects, and behavior-modifier influence of a range of EOs have been demonstrated on stored product pests [14,15,16,17].

Natural extracts from *Asarum* (Aristolochiaceae) plants showed antifungal and antimicrobial activity against some phytopathogens [18,19]. The extracts were also proven to have larvicidal activity against important mosquito vectors [20,21], and to be toxicants and reproductive inhibitors of the potato tuber moth *Phthorimaea operculella* [22]. To date, reports about *Asarum* plants are mainly focused on its broad spectrum of antifungal and antimicrobial activity or the control of agriculture field pests and mosquito vectors. *Asarum heterotropoides* var. *mandshuricum* (Maxim.) Kitag, belonging to the Aristolochiaceae, is a perennial herb distributed in Northeastern China [23]. Since ancient times, radix and rhizomes of *A. heterotropoides* have served as a kind of traditional Chinese medicine named “Xixin” for analgesic and anti-allergic purposes [24]. Early in the Ming dynasty, people took advantage of its special fragrance to protect Radix Ginseng from pest damage during storage [25]. This is still common in traditional Chinese medical pharmacies. However, investigations on active compounds from *A. heterotropoides* against stored product insects are few. Moreover, the aerial part of *A. heterotropoide*, as an unconventional medicinal portion, tends to be neglected, as it did not make the best of plant resources.

For this work, the chemical compositions of ER and EL from *A. heterotropoides* were compared. EOs from two different organs were evaluated for their toxicity and repellent activity against *L. serricorne* and *L. bostrychophila*.

## 2. Results

### 2.1. Chemical Composition of the Essential Oils

The yields of ER and EL were 5.01% and 4.85% (*v*/*w*), with densities of 1.05 and 0.97 g/mL respectively. Gas chromatography-mass spectrometry (GC-MS) analysis results are summarized in Table 1. The main components of ER included methyleugenol (28.67%), safrole (19.61%), 3,5-dimethoxytoluene (12.63%), and 3-carene (6.13%), whereas methyleugenol (27.05%), safrole (15.73%), α-pinene (6.84%) and 3,5-dimethoxytoluene (6.37%) were the main constituents of EL. EOs from *A. heterotropoides* were rich in phenylpropenes (>50%) and bicyclic monoterpenes.

### 2.2. Fumigant and Contact Toxicity

Results of fumigant and contact toxicity are presented in Table 2 and Table 3, respectively. ER and EL exhibited different levels of fumigant toxicity against *L. serricorne* (LC_50_ = 3.78 and 10.47 mg/L air, respectively). Safrole and 3,5-dimethoxytoluene were strongly toxic to *L. serricorne* and *L. bostrychophila* by fumigation (LC_50_ = 18.93 and 4.99 mg/L air; 0.83 and 0.91 mg/L air). Methyleugenol (LC_50_ = 10.82 mg/L air) showed similar fumigant activity with EL against *L. serricorne.* According to LD_50_ values, *L. serricorne* was more susceptible to EL (LD_50_ = 8.24 µg/adult) than ER (LD_50_ = 15.22 µg/adult), whereas *L. bostrychophila* displayed the opposite response. Methyleugenol, safrole, and 3,5-dimethoxytoluene were all observed to have potent contact toxicity against *L. serricorne* and *L. bostrychophila*. Notably, LD_50_ values of 3,5-dimethoxytoluene and safrole against *L. bostrychophila* were close to that of phosphine (LD_50_ = 18.72 µg/cm^2^). In previous papers, the contact toxicities of methyleugenol and safrole (LD_50_ = 12.8 and 14.6 µg/adult, respectively) against *L. serricorne* [26] were reported, as well as the fumigant and contact toxicity of methyleugenol (LC_50_ = 92.21 µg/L air and LD_50_ = 103.22 µg/cm^2^) against *L. bostrychophila* [27]. Here, the two compounds were retested. The effects were similar but LC_50_ or LD_50_ values differed quantitatively.

### 2.3. Repellent Activity

**Figure 1:**
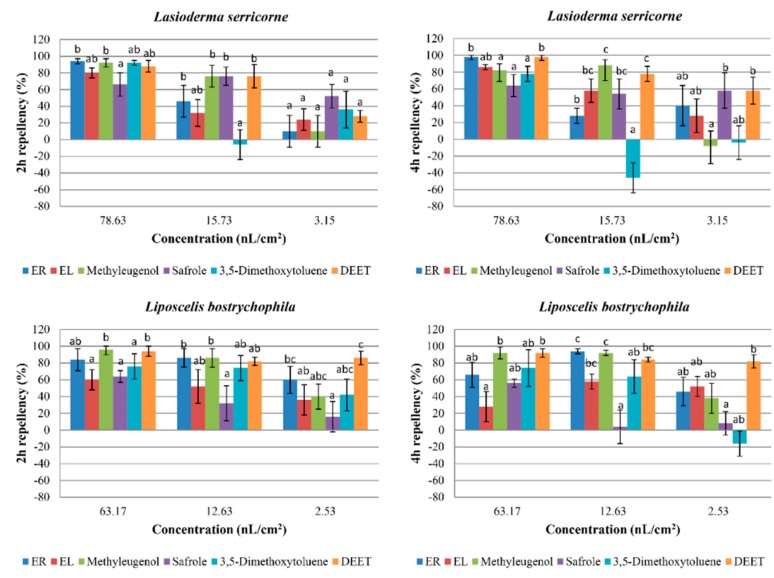
Percentage repellency (PR) of *Asarum heterotropoides* essential oil (EO) against *Lasioderma serricorne* and *Liposcelis bostrychophila* at 2 h and 4 h post-exposure. Means in the same column followed by the same letters do not differ significantly (*p* > 0.05) in ANOVA and Tukey’s tests. PR was subjected to an arcsine square-root transformation before ANOVA and Tukey’s tests. ER and EL: the essential oils from roots and leaves of *A. heterotropoides*, respectively.

The repellent results are presented in Figure 1. At the highest concentration, the percentage repellency (PR) of ER was higher than that of EL against the two insects. ER, EL, and methyleugenol demonstrated good repellent activity against *L. serricorne* at 78.63 nL/cm^2^ at two and four hours post-exposure. However, methyleugenol showed attractive effects on *L. serricorne* at 3.15 nL/cm^2^ at four hours post-exposure. 3,5-dimethoxytoluene showed effective repellency at 78.63 nL/cm^2^ at two hours post-exposure (PR = 92%) against *L. serricorne.* Methyleugenol also exhibited certain repellency against *L. bostrychophila* (PR > 85%) at 63.17 and 12.63 nL/cm^2^ at four hours post-exposure. 

## 3. Discussion

Methyleugenol, safrole, and 3,5-dimethoxytoluene were the most abundant compounds in ER in this work. The main components were consistent with those reported in some previous investigations of *A. heterotropoides* [20,29,30]. According to GC-MS analysis, *the* major components of ER and EL were methyleugenol and safrole, but minor components had some differences between the two organs. Content and distribution of individual constituents in EOs were affected by physiological diversities in tissue, organs, and metabolic pathways [31]. Methyleugenol and safrole were major volatile phenylpropenes in the EO of *A. heterotropoides*. Dan et al. [18] reported that methyleugenol largely contributes to the antimicrobial bioactivity of the EO. Otherwise, it was previously reported that methyleugenol had acaricidal activity toward a stored-food mite, *Tyrophagus putrescentiae* [32], and safrole was highly toxic to some mosquito larvae [20].

The phenylpropenes derivatives, safrole and methyleugenol, are good toxicants or repellents to a variety of pests, including *Sitophilus zeamais*, *Tribolium castaneum* [33], *Sitophilus oryzae* [34], *Periplaneta americana* [35], *Spodoptera litura* [36], *Blattella germanica* [37,38], etc. They were generally more effective than terpenes, such as limonene, cineole, and p-cymene. It was thought that methylenedioxy (-OCH_2_O-) could greatly enhance the insecticidal activity of phenylpropenes. The distance between the side chain double bond and the benzene ring, along with the methoxy groups attached to either the aromatic ring or the alkyl side chain of C_6_–C_3_ systems, appeared to be determining factors of their insecticidal efficacy and repellency [36,37]. Methyleugenol is a male attractant for certain *Bactrocera* species (Diptera), especially of the oriental fruit fly *Bactrocera dorsalis* (Hendel). Methyleugenol has been used as a part of the eco-friendly lure-and-kill approach in the control of fruit fly populations [39,40]. Here, methyleugenol showed repellency at high concentrations but attraction at lower concentrations against *L. serricorne.* However, *L. bostrychophila* was repelled by methyleugenol at all testing concentrations. Diptera and Coleoptera insects might be more likely to be attracted by low-concentration methyleugenol. This distinct phenomenon was largely related to insect species and sample concentrations. Further work is needed to clarify this finding. As is known, safrole is a hepatic carcinogen and methyleugenol is considered genotoxic. Thus, besides effectiveness evaluation, safety issues on mammalian health, non-targets, and the environment should be emphasized for promoting the practical application of natural products as novel pesticides [31,34,36,41]. Additionally, in this work, both adults of *L. serricorne* and *L. bostrychophila* exposed to safrole were observed to be inactive and torpid at the highest concentration, which could be related to mechanisms of neurotoxic actions [42,43]. Many studies showed neurotoxic actions of some EO components. In insects, they can cause paralysis followed by death. There are different mechanisms of action in EOs, such as the inhibition of acetylcholinesterase (AChE), the positive allosteric modulation of GABA, and competition with octopamine in binding to its receptor [44]. The exploration of mechanisms remains the focus of future work.

3,5-dimethoxytoluene is one of characteristic scent compounds emitted from flowers of most rose varieties [45]. Here, for the first time, 3,5-dimethoxytoluene was found to have significant toxicity and certain repellency against *L. serricorne* and *L. bostrychophila*. 3,5-Dimethoxytoluene, methyleugenol, and safrole are all oxygen-containing aromatic compounds. Among them, methyleugenol and safrole are phenylpropenes. The three testing compounds were potent toxicants against the two stored product insects. The EL of *A. heterotropoides* was confirmed to have insecticidal activity and repellency against *L. serricorne* and *L. bostrychophila*. This provides some scientific basis for comprehensive development and utilization of *A. heterotropoides* plant resources.

## 4. Materials and Methods

### 4.1. Chemicals

C_5_–C_36_ n-alkanes were purchased from Sigma-Aldrich, St. Louis, MO, USA. *N*,*N*-diethyl-3-methylbenzamide (DEET) was purchased from the National Center of Pesticide Standards, Shenyang, China. Methyleugenol was purchased from TCI (Shanghai) Development Co., Ltd., Shanghai, China. 3,5-dimethoxytoluene was purchased from Alfa Aesar (Tianjin) Chemical Co., Ltd., Tianjin, China. Safrole was isolated from a safrole-rich essential oil of *Michelia hedyosperma* Lew (Xishuangbanna, Yunnan Province, China). Fluon was purchased from Beijing Sino-Rich Material Science Co., Ltd., Beijing, China.

### 4.2. Plant Material

Roots and leaves of *A. heterotropoides* were collected from Liaoning Province, China in August 2013. Identification of the collections were verified by Dr. Q.R., Liu (College of Life Sciences, Beijing Normal University, Beijing, China) and the voucher specimens (BNU-CMH-Dushushan-2013-08-01-001; BNU-CMH-Dushushan-2013-08-01-002) were deposited in the Herbarium (BNU) of the College of Resources Science and Technology, Faculty of Geographical Science, Beijing Normal University.

### 4.3. Essential Oil Extraction

The fresh roots and leaves of *A. heterotropoides* were air-dried at room temperature and weighed separately. The two separated organs were subjected to hydrodistillation for 6 h to produce essential oils. The distilled oils were dehydrated with anhydrous sodium sulfate after extraction and their volumes were recorded for yield calculation. The two oil samples were stored in airtight containers in a refrigerator at 4 °C until use for bioassays.

### 4.4. Insects

The two species of insects were sampled from laboratory colonies. They were maintained in dark incubators at 28–30 °C and 70–80% relative humidity. *L. serricorne* were reared on wheat flour at 12–13% moisture content mixed with yeast (10:1, *w*/*w*). *L. bostrychophila* were bred on a mixture of milk powder, active yeast, and flour (1:1:10, *w*/*w*/*w*). The mixed-sex adults about 1 week old were used in all bioassays.

### 4.5. GC-FID and GC-MS Analysis

Components of two samples were determined by gas chromatography-flame ionization detector (GC-FID) and GC-MS on a Thermo Finnigan Trace DSQ instrument (Thermo Finnigan, Lutz, FL, USA) with a capillary column of HP-5MS (30 m × 0.25 mm × 0.25 μm stationary phase thickness). Similar testing conditions were used for both GC-FID and GC-MS. The column temperature was held at 50 °C for 2 min, then ramped up from 50 to 150 °C at 2 °C/min and held 2 min, and finally reached 250 °C at 10 °C/min, followed by a final hold at 250 °C for 5 min. The injector temperature was maintained at 250 °C. The volume injected was 1 μL of 1% solution (*v*/*v*, diluted in *n*-hexane). Helium was used as the carrier gas at a constant flow of 1 mL/min. Mass spectra were scanned from 50 to 550 *m*/*z* in full scan mode. Most constituents were identified by comparison of their retention indices with those taken from Adams (2001) and NIST 05 (Standard Reference Data, Gaithersburg, MD, USA). Retention indices (RI) were determined by comparison to a homologous series of *n*-alkanes (C_5_–C_36_) analyzed under the same operating conditions. Further identification was performed by comparing mass spectra to those in the NIST 05 and Wiley 275 libraries (Wiley, New York, NY, USA) or with those reported in the literature [46]. Relative percentages of individual components were obtained by averaging the GC-FID peak areas.

### 4.6. Fumigant Toxicity

The fumigant toxicity of EOs and individual compounds against *L. serricorne* were tested as described by Liu and Ho [47]. Preliminary experiments were conducted to determine appropriate ranges of testing concentrations. Serial dilution of all samples were prepared in *n*-hexane (five concentrations). Filter paper (diameter 2 cm) was impregnated with 10 μL of testing solution and each paper was placed on the underside of the screw cap of a glass vial (diameter 2.5 cm, height 5.5 cm, volume 27.5 mL). Ten adults were introduced into each vial and the cover was tightened immediately after processing. Fluon was properly coated inside each glass vial to prevent insect contact with the treated filter paper. As for booklice, the experimental method was described by Zhou et al. [48]. A filter paper strip (3.5 × 1.5 cm) was treated with 10 μL of an appropriate concentration of Eos or compounds. The impregnated filter paper was placed in the bottom of a glass jar (250 mL). Ten booklice in a glass bottle (8 mL) were placed into the larger glass jar and exposed for 24 h. For the two insect species, *n*-hexane was used as the negative control. Five replicates were performed for each treatment and control. The number of dead insects was counted after 24 h.

### 4.7. Contact Toxicity

For the evaluation of contact toxicity against *L. serricorne*, the method from Liu and Ho [47] was employed. Pre-experiments were carried out to define the appropriate testing concentrations. A serial dilution of Eos and compounds with five concentrations were dissolved in *n*-hexane to prepare. Aliquots (0.5 µL) of the dilutions were applied topically to the dorsal thorax of each insect. Ten insects were used for each concentration. The 10 treated insects were then transferred to each glass vial and kept in incubators. For the bioassays of contact toxicity against *L. bostrychophila*, refer to the method reported by Zhou et al. [48]. A filter paper (diameter 5.5 cm) was soaked with 300 μL of each testing solution. After the solvent was evaporated, the filter paper was fixed on the bottom of a Petri dish (diameter 5.5 cm). Ten booklice were placed into each Petri dish. Then, the dishes were covered and kept in incubators. For the two insect species, *n*-hexane was used as the negative control. All treatments and controls were replicated five times. The number of dead insects was counted after 24 h.

### 4.8. Repellent Test

The repellent activity of EOs and individual compounds was investigated according to the reference [49]. Petri dishes were used to confine insects here. For *L. serricorne*, the testing solutions of EOs/compounds with three concentrations (78.63, 15.73, and 3.15 nL/cm^2^) were prepared in *n*-hexane. *n*-hexane was used as the negative control and DEET was the positive control. The filter paper (9 cm in diameter) was cut in half. Each concentration with 500 μL was applied separately to a half-filter paper with a micropipette, as uniformly as possible. The other half was treated with an equal volume of *n*-hexane. The two halves were air-dried to evaporate the solvent and stuck together in a Petri dish. As for booklice, the Petri dishes and filter papers were 5.5 cm in diameter and the concentrations of EOs/compounds were set at 63.17, 12.63, and 2.53 nL/cm^2^. Two halves of a filter paper were treated with 150 μL of the solution. In all bioassays, 20 insects were released in the center of each disc and a cover was placed over the dish. Five replicates were carried out for each concentration. The number of insects present on the treated and control portions of each filter paper were recorded at 2 and 4 h post-exposure.

### 4.9. Statistical Analysis

All data were analyzed using SPSS V20.0 (IBM, New York, NY, USA). In the fumigant and contact assays, LC_50_ and LD_50_ values were calculated with Probit analysis [50]; 95% CI (Confidence interval), related parameters and chi-square values were estimated. In the repellent assays, the percent repellency (PR) was determined by the following equation:

PR (%) = [(*Nc* − *Nt*)/(*Nc* + *Nt*)] × 100

where *Nc* is the number of insects on the control half and *Nt* is the number of insects on the treated half. Percentage repellency values were transformed into arcsine and square root values for one-way ANOVA followed by Tukey’s test, respectively. Differences between means were considered significant when *p* < 0.05.

## 5. Conclusions

ER and EL from *A. heterotropoides* showed toxicity and repellency against *L. serricorne* and *L. bostrychophila.* EL of *A. heterotropoides* was verified to have certain toxicity and repellency against stored product insects, providing some scientific basis for comprehensive development and utilization of *A. heterotropoides* plant resources. Methyleugenol, safrole, and 3,5-dimethoxytoluene all exerted good insecticidal effects on *L. serricorne* and *L. bostrychophila*. *A. heterotropoides*, as a medicinal plant, has the potential to be applied in bio-insecticides. However, safety issues in terms of mammalian health, non-targets, and the environment remains to be ascertained in future work.

## Figures and Tables

**Table 1:** Chemical constituents identified from essential oils from roots (ER) and leaves (EL) of *Asarum heterotropoides*.

No.	Compound	RI Exp. ^a^	RI lit. ^a^	Relative Content (%)		Identified Method ^c^
				ER ^b^	EL ^b^	
**1**	Tricyclene	926	926	-	0.27	MS; RI
**2**	3-Thujene	934	929	0.20	0.17	MS; RI
**3**	*α*-Pinene	939	940	4.26	6.84	MS; RI
**4**	Camphene	949	952	0.83	5.49	MS; RI
**5**	*β*-Pinene	978	975	5.13	6.03	MS; RI
**6**	*α*-Phellandrene	1007	1004	1.08	0.28	MS; RI
**7**	3-Carene	1011	1010	6.13	4.40	MS; RI
**8**	o-Cymol	1020	1022	0.79	-	MS; RI
**9**	m-Cymol	1023	1025	-	1.08	MS; RI
**10**	Sylvestrene	1027	1028	0.96	-	MS; RI
**11**	Limonene	1032	1032	-	0.73	MS; RI
**12**	Eucalyptol	1046	1037	0.64	0.75	MS; RI
**13**	*γ*-Terpinene	1056	1059	0.16	-	MS; RI
**14**	Terpinolene	1093	1095	0.31	0.19	MS; RI
**15**	Camphor	1146	1146	0.14	0.37	MS; RI
**16**	Borneol	1166	1171	0.88	4.70	MS; RI
**17**	Terpinen-4-ol	1174	1177	0.29	0.23	MS; RI
**18**	*α*-Terpinol	1190	1189	0.43	0.36	MS; RI
**19**	Estragole	1198	1200	0.64	0.56	MS; RI
**20**	Methyl thymyl ether	1235	1232	-	0.20	MS; RI
**21**	Eucarvone	1243	1245	2.58	1.92	MS; RI
**22**	3,5-Dimethoxytoluene	1269	1260	12.63	6.37	MS; RI
**23**	Safrole	1289	1289	19.61	15.73	MS; RI
**24**	3,4,5-Trimethoxytoluene	1408	1408	5.10	2.90	MS; RI
**25**	Methyleugenol	1410	1407	28.67	27.05	MS; RI
**26**	*β*-Gurjunene	1428	1424	0.25	0.63	MS; RI
**27**	*β*-Cedrene	1432	1428	-	0.15	MS; RI
**28**	(Z)-*β*-Farnesene	1442	1445	-	1.82	MS; RI
**29**	*α*-Patchoulene	1457	1456	-	0.12	MS; RI
**30**	Allo-Aromadendren	1467	1461	-	0.22	MS; RI
**31**	Myristicin	1519	1519	4.88	5.53	MS; RI
**32**	Elemicin	1558	1554	0.41	2.80	MS; RI
**33**	Caryophylene oxide	1561	1561	-	0.18	MS; RI
**34**	Humulene epoxide II	1606	1609	-	0.15	MS; RI
**35**	Patchouli alcohol	1657	1657	-	0.41	MS; RI
	Phenylpropenes			54.21	51.67	
	Monoterpenes			24.02	32.73	
	Total			96.98	98.62	

^a^ RI exp.: retention index as determined on a HP-5MS capillary column using the homologous series of n-alkanes; RI lit.: retention index taken from the NIST 05 library; ^b^ ER and EL: the essential oils from roots and leaves of *A. heterotropoides,* respectively; ^c^ MS: based on comparison of mass spectra with those listed in the NIST 05 and Wiley 275 libraries or with those reported in the literatures; “-” means “not be identified”.

**Table 2:** Fumigant toxicity of *A. heterotropoides* essential oil against *L. serricorne* (LS) and *L. bostrychophila* (LB).

Insect	Treatment	Concentration (%) ^a^	LC_50_ (mg/L air) (95% LCL-UCL ^b^)	Slope ± SE	Chi Square (χ^2^)	*p*-Value
LS	ER ^c^	5.00–0.99	3.78 (2.53–5.04)	2.63 ± 0.45	10.96	0.984
	EL ^c^	10.00–1.98	10.47 (8.53–11.63)	3.37 ± 0.46	12.07	0.969
	Methyleugenol	10.00–1.98	10.82 (7.07–13.73)	1.78 ± 0.35	13.03	0.951
	Safrole	15.00–2.96	18.93 (13.39–23.68)	2.24 ± 0.48	12.86	0.710
	3,5-Dimethoxytoluene	4.44–0.88	4.99 (4.57–5.82)	4.11 ± 0.51	7.23	0.999
	Phosphine ^d^	-	9.23 (7.13–11.37) × 10^−3^	2.12 ± 0.27	11.96	0.971
LB	ER ^c^	10.00–1.98	1.42 (1.25–1.59)	5.32 ± 0.74	6.77	0.913
	EL ^c^	10.00–1.98	1.73 (1.53–1.96)	4.77 ± 0.64	10.10	0.686
	Methyleugenol	0–50	-	-	-	-
	Safrole	5.00–0.98	0.83 (0.74–0.94)	5.07 ± 0.69	3.97	0.991
	3,5-Dimethoxytoluene	5.00–0.98	0.91 (0.82–1.01	6.35 ± 0.87	7.37	0.882
	Dichlorvos ^e^	-	1.35 (1.08–1.62) × 10^−3^	8.71 ± 0.65	9.78	0.926

^a^ Concentrations: ranges of testing concentrations in the experiment; ^b^ LCL-UCL: lower confidence limit—upper confidence limit; ^c^ ER and EL: the essential oils from roots and leaves of *A. heterotropoides* separately; ^d^ Data from Yang et al. [28]; ^e^ Data from Liu et al. [27].

**Table 3:** Contact toxicity of *A. heterotropoides* essential oil against *L. serricorne* (LS) and *L. bostrychophila* (LB).

Insect	Treatment	Concentrations (%) ^a^	LD_50_ (µg/adult)/(µg/cm^2^) (95% LCL-UCL ^b^)	Slope ± SE	Chi Square (χ^2^)	*p*-Value
LS	ER ^c^	6.67–1.32	15.22 (13.65–17.32)	5.89 ± 0.67	13.98	0.927
	EL ^c^	4.44–0.88	8.24 (7.75–9.21)	9.09 ± 0.94	15.11	0.891
	Methyleugenol	5.00–0.99	12.71 (11.16–14.59)	3.08 ± 0.38	10.12	0.991
	Safrole	4.44–0.88	9.08 (7.55–10.75)	3.10 ± 0.50	7.86	0.853
	3,5-Dimethoxytoluene	10.00–1.98	23.73 (19.52–28.86)	2.72 ± 0.47	11.88	0.537
	Pyrethrins ^d^	-	0.24 (0.16–0.35)	1.31 ± 0.20	17.36	0.791
LB	ER ^c^	0.50–0.24	38.86 (36.87–40.71)	5.31 ± 0.60	13.24	0.947
	EL ^c^	0.67–0.32	52.01 (49.57–54.34)	3.63 ± 0.39	11.61	0.976
	Methyleugenol	0.44–0.17	31.66 (29.69–34.02)	8.49 ± 1.12	12.41	0.715
	Safrole	0.20–0.10	21.32 (20.07–22.70)	9.08 ± 1.12	6.86	0.976
	3,5-Dimethoxytoluene	0.20–0.10	18.63 (17.45–19.82)	10.71 ± 1.39	6.78	0.913
	Pyrethrins ^d^	-	18.72 (17.60–19.92)	2.98 ± 0.40	10.56	0.987

^a^ Concentrations: ranges of testing concentrations in the experiment; ^b^ LCL-UCL: lower confidence limit—upper confidence limit; ^c^ ER & EL: the essential oils from roots and leaves of *A. heterotropoides* separately; ^d^ Data from Yang et al. [28].

## References

[1] Maxmen A. (2013). Crops pests: Under attack. Nature.

[2] Papadopoulou S.C., Buchelos C.T. (2002). Definition of the flight period of *Lasioderma serricorne* (F.) in stored tobacco. J. Pestic. Sci..

[3] Howard R.W., Lord J.C. (2003). Cuticular lipids of the booklouse, *Liposcelis bostrychophila*: Hydrocarbons, aldehydes, fatty acids, and fatty acid amides. J. Chem. Ecol..

[4] Sağlam O., Edde P.A., Phillips T.W. (2015). Resistance of *Lasioderma serricorne* (Coleoptera: Anobiidae) to fumigation with phosphine. J. Econ. Entomol..

[5] Ishibashi O., Sakuragi K., Fukutomi Y., Kawakami Y., Kamata Y., Sakurai M., Nakayama S., Uchiyama H., Kobayashi H., Kojima H. (2017). Lip b 1 is a novel allergenic protein isolated from the booklouse, *Liposcelis bostrychophila*. Allergy.

[6] Li M., Li X.J., Lü J.H., Huo M.F. (2018). The effect of acclimation on heat tolerance of *Lasioderma serricorne* (Fabricius) (Coleoptera: Anobiidae). J. Therm. Biol..

[7] Boyer S., Zhang H., Lempérière G. (2012). A review of control methods and resistance mechanisms in stored-product insects. Bull. Entomol. Res..

[8] Antwi F.B., Reddy G.V.P. (2015). Toxicological effects of pyrethroids on non-target aquatic insects. Environ. Toxicol. Pharmacol..

[9] Roy D.N., Goswami R., Pal A. (2017). The insect repellents: A silent environmental chemical toxicant to the health. Environ. Toxicol. Pharmacol..

[10] Wijayaratne L.K.W., Arthur F.H., Whyard S. (2018). Methoprene and control of stored-product insects. J. Stored Prod. Res..

[11] Isman M.B. (2006). Botanical insecticides, deterrents, and repellents in modern agriculture and an increasingly regulated world. Annu. Rev. Entomol..

[12] Rajendran S., Sriranjini V. (2008). Plant products as fumigants for stored-product insect control. J. Stored Prod. Res..

[13] Caballero-Gallardo K., Olivero-Verbel J., Stashenko E.E. (2011). Repellent activity of essential oils and some of their individual constituents against *Tribolium castaneum* Herbst. J. Agric. Food Chem..

[14] Isman M.B. (2000). Plant essential oils for pest and disease management. Crop Prot..

[15] Miresmailli S., Isman M.B. (2014). Botanical insecticides inspired by plant-herbivore chemical interactions. Trends Plant Sci..

[16] Raut J.S., Karuppayil S.M. (2014). A status review on the medicinal properties of essential oils. Ind. Crops Prod..

[17] Quan M.R., Liu Q.Z., Liu Z.L. (2018). Identification of insecticidal constituents from the essential oil from the aerial parts *Stachys riederi var. japonica*. Molecules.

[18] Dan Y., Liu H.Y., Gao W.W., Chen S.L. (2010). Activities of essential oils from *Asarum heterotropoides* var. mandshuricum against five phytopathogens. Crop Prot..

[19] Wang G.Q., Zhang T. (2011). The effect of Asarum essential oils on the hyphae membrane permeability of *Botrytis cinerea*. Acta Agric. Boreali-Sin..

[20] Perumalsamy H., Kim N.J., Ahn Y.J. (2009). Larvicidal activity of compounds isolated from *Asarum heterotropoides* against *Culex pipiens pallens*, *Aedes aegypti*, and *Ochlerotatus togoi* (Diptera: Culicidae). J. Med. Entomol..

[21] Liu Y., Ji L.Z., Wang G.Q., Wang Y.X., Zhang Y., Yi X.M. (2014). Impact of Asarum essential oils on physiological and biochemical indicators of two agricultural pests. Plant Dis. Pests.

[22] Wu M., Xiong Y., He Q.Z., Li K.Z., Xiao C. (2017). Prevention and control effect of *Asarum sieboldii* fumigation against *Phthorimaea operculella* during potato storage. Guizhou Agric. Sci..

[23] Huang S.M., Kelly L.M., Gilbert M.G. (2003). Aristolochiaceae. Flora China.

[24] Jing Y., Zhang Y.F., Shang M.Y., Liu G.X., Li Y.L., Wang X., Cai S.Q. (2017). Chemical constituents from the roots and rhizomes of *Asarum heterotropoides* var *mandshuricum* and the in vitro anti-inflammatory activity. Molecules.

[25] Chen J.M. (2009). Ben Cao Meng Qvan.

[26] Du S.S., Yang K., Wang C.F., You C.X., Geng Z.F., Guo S.S., Deng Z.W., Liu Z.L. (2014). Chemical constituents and activities of the essential oil from *Myristica fragrans* against cigarette beetle *Lasioderma serricorne*. Chem. Biodivers..

[27] Liu X.C., Zhou L.G., Liu Z.L., Du S.S. (2013). Identification of insecticidal constituents of the essential oil of *Acorus calamus* rhizomes against *Liposcelis bostrychophila* Badonnel. Molecules.

[28] Yang K., Wang C.F., You C.X., Geng Z.F., Sun R.Q., Guo S.S., Du S.S., Liu Z.L., Deng Z.W. (2014). Bioactivity of essential oil of *Litsea cubeba* from China and its main compounds against two stored product insects. J. Asia Pac. Entomol..

[29] Wang B.B., Qi W., Wang L.L., Kong D.Q., Kano Y., Li J.X., Yuan D. (2014). Comparative study of chemical composition, antinociceptive effect and acute toxicity of the essential oils of three Asarum drugs. J. Chin. Pharm. Sci..

[30] Kim J.R., Perumalsamy H., Lee J.H., Ahn Y.J., Lee Y.S., Lee S.G. (2016). Acaricidal activity of *Asarum heterotropoides* root-derived compounds and hydrodistillate constitutes toward *Dermanyssus gallinae* (Mesostigmata: Dermanyssidae). Exp. Appl. Acarol..

[31] Guo S.S., Geng Z.F., Zhang W.J., Liang J.Y., Wang C.F., Deng Z.W., Du S.S. (2016). The chemical composition of essential oils from *Cinnamomum camphora* and their insecticidal activity against the stored product pests. Int. J. Mol. Sci..

[32] Lee C.H., Sung B.K., Lee H.S. (2006). Acaricidal activity of fennel seed oils and their main components against *Tyrophagus putrescentiae*, a stored-food mite. J. Stored Prod. Res..

[33] Huang Y., Ho S.H., Kini R.M. (1999). Bioactivities of safrole and isosafrole on *Sitophilus zeamais* (Coleoptera: Curculionidae) and *Tribolium castaneum* (Coleoptera: Tenebrionidae). J. Econ. Entomol..

[34] Kim J., Park I.K. (2010). Fumigant toxicity of Korean medicinal plant essential oils and components from *Asiasarum sieboldi* root against *Sitophilus oryzae* L.. Flavour Frag. J..

[35] Ngoh S.P., Choo L.E.W., Pang F.Y., Huang Y., Kini M.R., Ho S.H. (1998). Insecticidal and repellent properties of nine volatile constituents of essential oils against the American cockroach, *Periplaneta americana* (L.). J. Pestic. Sci..

[36] Bhardwaj A., Tewary D.K., Kumar R., Kumar V., Sinha A.K., Shanker A. (2010). Larvicidal and structure-activity studies of natural phenylpropanoids and their semisynthetic derivatives against the tobacco armyworm *Spodoptera litura* (fab.) (lepidoptera: Noctuidae). Chem. Biodivers..

[37] Jung W.C., Jang Y.S., Hieu T.T., Lee C.K., Ahn Y.J. (2007). Toxicity of *Myristica fagrans* seed compounds against *Blattella germanica* (dictyoptera: Blattellidae). J. Med. Entomol..

[38] Yeom H.J., Kang J., Kim S.W., Park I.K. (2013). Fumigant and contact toxicity of Myrtaceae plant essential oils and blends of their constituents against adults of German cockroach (*Blattella germanica*) and their acetylcholinesterase inhibitory activity. Pestic. Biochem. Phys..

[39] Khrimian A., Jang E.B., Nagata J., Carvalho L. (2006). Consumption and metabolism of 1,2-dimethoxy-4-(3-fluoro-2-propenyl) benzene, a fluorine analog of methyl eugenol, in the oriental fruit fly *Bactrocera dorsalis* (Hendel). J. Chem. Ecol..

[40] Haq I., Cáceres C., Meza J.S., Hendrichs J., Vreysen M.J.B. (2018). Different methods of methyl eugenol application enhance the mating success of male oriental fruit fly (Dipera: Tephritidae). Sci. Rep..

[41] Wang Y., Dai P.P., Guo S.S., Cao J.Q., Pang X., Geng Z.F., Sang Y.L., Du S.S. (2018). Supercritical carbon dioxide extract of *Cinnamomum cassia* bark: Toxicity and repellency against two stored-product beetle species. Environ. Sci. Pollut. Res..

[42] Pajaro-Castro N., Caballero-Gallardo K., Olivero-Verbel J. (2017). Neurotoxic effects of linalool and *β*-pinene on *Tribolium castaneum* Herbst. Molecules.

[43] Cao J.Q., Guo S.S., Wang Y., Pang X., Geng Z.F., Du S.S. (2018). Toxicity and repellency of essential oil from *Evodia lenticellata* Huang fruits and its major monoterpenes against three stored-product insects. Ecotoxicol. Environ. Saf..

[44] Jankowska M., Rogalska J., Wyszkowska J., Stankiewicz M. (2018). Molecular targets for components of essential oils in the insect nervous system—A review. Molecules.

[45] Chen X., Baldermann S., Cao S., Lu Y., Liu C., Hirata H., Watanabe N. (2015). Developmental patterns of emission of scent compounds and related gene expression in roses of the cultivar *Rosa xhybrida* cv. ‘Yves Piaget’. Plant Physiol. Biochem..

[46] Adams R.P. (2001). Identification of Essential Oil Components by Gas Chromatography/Quadrupole Mass Spectroscopy.

[47] Liu Z.L., Ho S.H. (1999). Bioactivity of the essential oil extracted from *Evodia rutaecarpa* Hook f. et Thomas against the grain storage insects, *Sitophilus zeamais* Motsch. and *Tribolium castaneum* (Herbst). J. Stored Prod. Res..

[48] Zhou H.Y., Zhao N.N., Du S.S., Yang K., Wang C.F., Liu Z.L., Qiao Y.J. (2012). Insecticidal activity of the essential oil of *Lonicera japonica* flower buds and its main constituent compounds against two grain storage insects. J. Med. Plants Res..

[49] Zhang J.S., Zhao N.N., Liu Q.Z., Liu Z.L., Du S.S., Zhou L.G., Deng Z.W. (2011). Repellent constituents of essential oil of *Cymbopogon distans* aerial parts against two stored-product insects. J. Agric. Food Chem..

[50] Sakuma M. (1998). Probit analysis of preference data. Appl. Entomol. Zool..

